# *Trypanosoma cruzi* Immune Response Modulation Decreases Microbiota in *Rhodnius prolixus* Gut and Is Crucial for Parasite Survival and Development

**DOI:** 10.1371/journal.pone.0036591

**Published:** 2012-05-04

**Authors:** Daniele P. Castro, Caroline S. Moraes, Marcelo S. Gonzalez, Norman A. Ratcliffe, Patrícia Azambuja, Eloi S. Garcia

**Affiliations:** 1 Laboratório de Bioquímica e Fisiologia de Insetos, Instituto Oswaldo Cruz, Fundação Oswaldo Cruz (Fiocruz), Rio de Janeiro, Rio de Janeiro, Brazil; 2 Laboratório de Biologia de Insetos, Departamento de Biologia Geral, Instituto de Biologia, Universidade Federal Fluminense (UFF) Niterói, Rio de Janeiro, Brazil; 3 Departamento de Entomologia Molecular, Instituto Nacional de Entomologia Molecular (INCT-EM), Rio de Janeiro, Rio de Janeiro, Brazil; Kansas State University, United States of America

## Abstract

*Trypanosoma cruzi* in order to complete its development in the digestive tract of *Rhodnius prolixus* needs to overcome the immune reactions and microbiota trypanolytic activity of the gut. We demonstrate that in *R. prolixus* following infection with epimastigotes of *Trypanosoma cruzi* clone Dm28c and, in comparison with uninfected control insects, the midgut contained (i) fewer bacteria, (ii) higher parasite numbers, and (iii) reduced nitrite and nitrate production and increased phenoloxidase and antibacterial activities. In addition, in insects pre-treated with antibiotic and then infected with Dm28c, there were also reduced bacteria numbers and a higher parasite load compared with insects solely infected with parasites. Furthermore, and in contrast to insects infected with Dm28c, infection with *T. cruzi* Y strain resulted in a slight decreased numbers of gut bacteria but not sufficient to mediate a successful parasite infection. We conclude that infection of *R. prolixus* with the *T. cruzi* Dm28c clone modifies the host gut immune responses to decrease the microbiota population and these changes are crucial for the parasite development in the insect gut.

## Introduction

Chagas disease is an endemic parasitic disease important in large areas of Latin America [1]. The majority of triatomine species found in this region are potential vectors for *Trypanosoma cruzi*, the causative agent of the disease [1], [2]. *Rhodnius prolixus* is the main vector of Chagaś disease in the northern part of South America and in some areas of Central America [3].

The *T. cruzi* – triatomine vector interactions are complex and mediated by numerous parasite and insect factors [4]–[8]. The parasite circulates between humans, domestic and sylvatic mammalian reservoirs, and insect vectors [6]–[8]. *T. cruzi* infection of the insect vector starts with the ingestion of an infective blood meal. A successful transmission depends upon the parasite multiplication and differentiation in the triatomine gut [7]–[9].

Many factors have been implicated in the establishment of *T. cruzi* infection in the insects vector gut including a crop lytic factor, lectins, gut enzymes and microbiota-derived factors, antimicrobial compounds and nitric oxide [4], [6]–[12]. In addition, the gut structural organization is important for parasite attachment to the perimicrovilar membranes and their subsequent development [5], [9], [13]. The success of parasite infection also depends upon the *T. cruzi* strain used and the insects species infected. For example, in *R. prolixus,* the *T. cruzi* Dm28c clone and CL strains are capable of completing their developmental cycles in contrast to the Y strain which rapidly disappears from the insect's gut [14], [15].

Regarding the role of the diverse gut microbiota in modulating *T. cruzi* development in the gut of *R. prolixus*, we have previously identified a *Serratia* sp. isolated from this insect vector which interferes with some strains of parasite development and lyses parasites in *in vitro* experiments [7], [14], [16]–[18]. The microbiota has also previously been shown to modulate the vectorial competence of the insect host by means of direct interaction with parasites or by competition for resources in the gut [7]–[11]. In addition, microbiota can constrain pathogen development indirectly by inducing vector antiparasitic activity and humoral immune defense factors in particular [8], [12], [16], [19]–[21].

Humoral defenses in insects are characterized by a battery of potent antimicrobial peptides (AMPs), reactive intermediates of nitrogen or oxygen, and complex enzymatic cascades, such as the prophenoloxidase system, that contribute to clotting or hemolymph melanization [22]. These humoral factors may be secreted directly into the hemocoel or into the gut lumen as components of the immune response to eliminate potential pathogens acquired during feeding [7], [8], [23].

In this paper, we have examined the relationship in the gut of *R. prolixus* between the microbiota and the insect's humoral antibacterial compounds with the level of infection following feeding with *T. cruzi*. In particular, we investigated the role of *T. cruzi* infection in the modulation of the microbiota population in the *R. prolixus* gut. We demonstrated, for the first time, that infection with *T. cruzi* Dm28c clone, but not with the Y strain, changes the microbiota population in the digestive tract by modulating the host immune responses and that this contributes to parasite development in the gut of *R. prolixus*.

## Results

### *Trypanosoma cruzi* infection and microbiota population in the digestive tract

**Figure 1:**
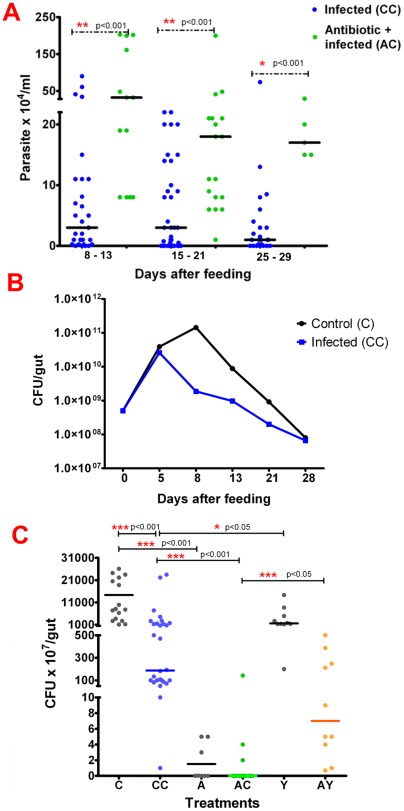
Parasite infection and microbiota population in *Rhodnius prolixus* 5^th^-instar nymphs digestive tract challenged by *Trypanosoma cruzi* Dm28c clone. (A) Parasite infection at different days after infection. (B) Microbiota population at different days after feeding. (C) Microbiota population 8 days after feeding. Treatments: C – control insects fed on blood alone; CC – insects infected with *T. cruzi* Dm28c clone; A – insects treated with antibiotic alone; AC- insects treated with antibiotic and infected with the Dm28c parasites; Y- insects infected with *T. cruzi* Y strain; AY–insects treated with antibiotic and infected with *T. cruzi* Y strain. In figures A and C each point represents the number of parasites or bacteria in an individual digestive tract, and horizontal lines indicate the median. In figure B each point represents the median. In figure C the median for antibiotic treated and infected insects (AC) is zero and therefore overlaps the x axes. Treatments were repeated 3–5 times with 6–10 insects in each experiment reaching a total of 25 to 35 insects for each group. Medians were analyzed with 1 way ANOVA and Mann Whitney test.

Parasite infection and microbiota population in *R. prolixus* 5^th^-instar nymphs whole digestive tract were analyzed from 5 to 29 days after treatment. In this period, the median number of *T. cruzi* Dm28c clone in the whole digestive tract (CC) ranged from 1 to 3×10^4^ parasites/ml (Figure 1A). Regarding the microbiota, the median number of gut bacteria in the control insects fed blood alone (C) showed a rapid increase and by 8 days after feeding reached a peak of 1.4×10^11^ before reducing to 8×10^7^ bacteria/digestive tract by day 28 after feeding (Figure 1B) and then remained stable in numbers until the next feeding. Significant differences in bacterial numbers between parasite-infected (CC) and control (C) groups were observed at 8 days after initiating the experiments. At this time in control insects, the microbiota reached 1.4×10^11^ bacteria (CFU) whereas parasite-infected insects presented only 1.8×10^9^ CFU /digestive tract (Figures 1B, 1C; p<0.001).

To investigate the role of the reduction of the microbiota upon *T. cruzi* Dm28c infection in *R. prolixus*, one group of 5^th^ instar nymphs was treated with antibiotic solution alone added to the blood meal (A), and another group of insects was treated with antibiotic and then infected with parasites (AC). With this non toxic dose of antibiotic in the blood meal, we observed an increase in *T. cruzi* Dm28c infection (AC), in comparison with insects fed with parasites alone (CC), ranging from 3.2×10^5^ parasites/ml to 1.7×10^5^ from 8–13 days to 25–29 days, respectively, after infective feeding (Figure 1A). Antibiotic treated and infected insects (AC) presented 10 times more parasites than in control infected insects (CC) at 8–13 days after feeding (Figure 1A; p<0.001). As expected, upon antibiotic treatment alone (A), the bacteria population was significantly reduced (p<0.001) compared with the controls (C) (Figure 1C). In the group of insects treated with antibiotic and infected with parasites (AC), we noted few bacteria in the digestive tract as in the group treated with antibiotic alone (A) (Figure 1C; p>0.1).

Also, to help to clarify whether the microbiota could be responsible for the low infection rates of other *T. cruzi* strains in *R. prolixus,* groups of insects were infected with *T. cruzi* Y strain and also treated with antibiotics. The *T. cruzi* Y strain, as expected, did not develop in *R. prolixus*, as previously demonstrated by Azambuja *et al.* [14]. The antibiotic-treated insects subsequently infected with *T. cruzi* Y strain, also failed to develop any parasite infections (not shown). A significant reduction was observed in the microbiota population in insects treated with *T. cruzi* Y strain (Y; median 1.6×10^10^ CFU) eight days after feeding compared with the control group (C; median 1.4×10^11^ CFU) (p<0.05). In contrast, a significantly higher microbiota was recorded in the group treated with *T. cruzi* Y strain (Y) in comparison with insects infected with *T. cruzi* Dm28c (CC; median 1.8×10^9^ CFU) (p<0.05) (Figure 1C). Antibiotic-treated insects infected with *T. cruzi* Y strain (AY; median 7×10^7^ CFU) also had significantly higher bacterial numbers than antibiotic-treated insects subsequently infected with Dm28c clone (AC; median 0 CFU) (Figure 1C; p<0.05).

### Bacterial growth inhibition

**Figure 2:**
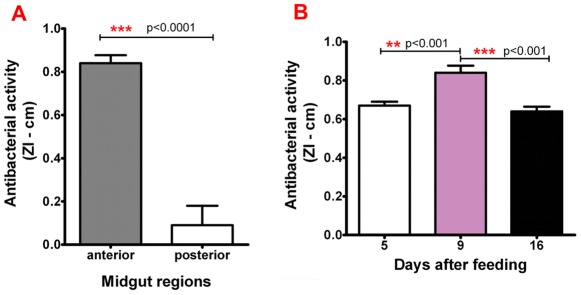
Antibacterial activity using inhibition zone (ZI) assay in *Rhodnius prolixus* 5^th^-instar nymphs infected with *Trypanosoma cruzi* Dm28c clone. (A) Antibacterial activity with anterior and posterior midgut regions at 9 days after feeding; (B) Antibacterial activity of anterior midgut at 5, 9 and 16 days after feeding. Bars represent mean ± SEM. Means were analyzed by using t Test and 1 way ANOVA. Each bar represents the mean (+/−SEM) of four separate experiment, n = 6−10 insects for each determination.

The anterior midgut antimicrobial activities at 9 days after feeding in the ZI assay (0.84±0.04 cm diameter) were significantly higher than posterior midgut (0.09±0.09 cm diameter) of control infected insects (CC) (Figure 2A; p<0.001). Therefore, the bacterial growth inhibition and immune reactions experiments were standardized using samples of anterior midgut. We also compared with the ZI assay, the antibacterial activities of the anterior midgut at days 5, 9 and 16 after infective feeding (CC), and observed that at day 9 the activity (0.84±0.04 cm diameter) was significantly higher compared with day 5 (0.67±0.02 cm diameter) and day 16 (0.64±0.02 cm diameter) (Figure 2B; p<0.001).

### Gut antimicrobial activities

In order to obtain a better understanding of the regulation of microbiota in the vector anterior midgut, the antimicrobial activities in the anterior midgut of control *R. prolixus* (C) and insects infected with *T. cruzi* Dm28c clone (CC) were investigated.

**Figure 3:**
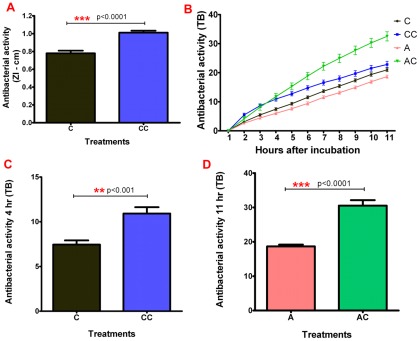
Antibacterial activity in *Rhodnius prolixus* 5^th^-instar nymphs challenged by *Trypanosoma cruzi* Dm28c clone. **Anterior midgut samples collected nine days after feeding and infection.** (A) Inhibition zone (ZI) assay incubated for 24 h at 37°C. (B) Turbidometric (TB) assay incubated for 11 h with readings at each hour. (C) Turbidometric (TB) assay after 4 h of incubation of uninfected or infected control insects. (D) Turbidometric assay after 11 h of incubation of insects treated with antibiotic alone or with antibiotic and then infected. Treatments: C – control insects fed on blood alone; CC – parasite infected insects; A – insects treated with antibiotic; AC- insects treated with antibiotic and then infected with parasites. Each bar represents mean ± SEM of four experiments, n = 6–10 insects for each determination. Means were analyzed by using t Test, Mann Whitney test and 2 way ANOVA.

The results of the ZI assays showed significantly smaller zones of inhibition (ZI) in control insects (C) (0.78 cm±0.03) when compared with control infected insects (CC) (1.0 cm±0.02) (Figure 3A; p<0.0001). The effect of *T. cruzi* Dm28c infection on antibacterial activities in the anterior midgut was also tested using a turbidometric assay (TB). Experiments incubating the extracts with bacteria for 11 h at 37°C showed significantly higher antibacterial activity in infected insects (CC) compared with control insects (C) only after 4, 5 and 6 h of incubation (Figure 3B; p<0.05). For example, parasite-infected insects (CC), after 4 h incubation, recorded an antibacterial activity of 10.92 (±0.71), significantly higher than control groups (C) with activity of 7.45 (±0.47) (Figure 3C; p = 0.001). These results were confirmed by following bacterial growth on BHI-agar plates where control (C) and infected insects (CC) presented low numbers of bacteria (not shown).

To test the hypothesis that the higher parasitemia and lower microbiota recorded in antibiotic-treated insects resulted from modulation of the antibacterial immune responses was also investigated further. ZI assays with insects treated just with antibiotics (A) or with antibiotics and then infected with parasites (AC) showed inhibition zones much bigger than with the non-antibiotic C and CC groups with diameters larger than 2.0 cm after 18 h but these could not be measured accurately as the zones overlapped with adjacent samples (not shown). In TB assays, the antibiotic-treated and infected insects (AC) had the highest activity of all groups and this activity was significantly higher from 4 to 11 h of incubation compared with antibiotic-treated insects (A) (Figure 3B; p<0.001). Comparing antibiotic-treated insects infected with clone Dm28c (AC) with antibiotic treated insects (A) after 11 h, a significant difference in antibacterial activity was recorded of 30.54 (±1.58) and 18.66 (±0.52), respectively (Figure 3D; p<0.0001).

### Phenoloxidase (PO) activities

**Figure 4:**
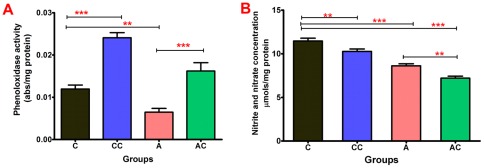
Phenoloxidase activity (A) and nitrite and nitrate production (B) in *Rhodnius prolixus* 5^th^-instar nymphs challenged by *Trypanosoma cruzi* Dm28c clone. Anterior midgut samples collected nine days after feeding and infection. Treatments: C – control insects fed on blood alone; CC – parasite infected insects; A – insects treated antibiotic; AC- insects treated with antibiotic and infected with the parasite. Bars represent mean ± SEM. Means were analyzed by using 1 way ANOVA comparing all groups to the control (C) and all groups to the insects treated with antibiotic (A). Each experiment represents the mean (+/−SEM) of four separate experiment, n = 6–10 insects for each determination; p<0.001, *** extremely significant, ** very significant and * significant.

PO activities were also measured in the gut of *R. prolixus* 5^th^ instar nymphs 9 days after feeding. PO specific activities were highest in the insects infected with *T. cruzi* Dm28c clone (CC) (0.024 abs/mg protein) compared with the control group (C) (0.012 abs/mg protein) (Figure 4A; p<0.001). Antibiotic-treated and infected insects (AC) recorded results (0.016 abs/mg protein) higher than insects treated with antibiotic alone (A) (0.006 abs/mg protein) but lower than the insects infected with *T. cruzi* Dm28c (CC) (Figure 4A; p<0.001).

### Nitrite/nitrate productions

The last immune reaction investigated for modulation by the *T. cruzi* Dm28c infection was the production of NO (nitrite/nitrate) in the gut. This activity showed a significant decrease in the infected group (CC) compared with the controls (C) and the levels varied from 10.3 μMols/mg protein to 11.5 μMols/mg protein, respectively (Figure 4B; p<0.001). In antibiotic-treated insects, NO (nitrite/nitrate) levels were also lower for infected insects so that in antibiotic-treated and infected insects (AC) 7.2 μMols/mg protein were recorded compared with 8.6 μMols/mg protein for the insects treated with antibiotic alone (A) (Figure 4B; p<0.001).

## Discussion

In triatomine insects, there is a close association of gut microorganisms with parasites. Different parasite species or strains infecting *R. prolixus* can increase or decrease the midgut bacterial flora. Thus, Eichler and Schaub [19] showed that *R. prolixus* infected with *T. rangeli* strain Choachi, but not with *T. cruzi* strain ‘Chile 5’, reduced the symbiont *Rhodococcus rhodnii* concentration by 50%. Furthermore, we demonstrate herein that *T. cruzi* Dm28c clone establishes a parasite infection and reduces *R. prolixus* microbiota while strain Y fails to infect *R. prolixus* and the microbiota remain at significantly higher levels than with the Dm28c clone. These observations on parasite and insect microbiota interactions are probably related to the success of parasite development in insects. The difference of our results from Eichler and Schaub may be related to different strains/clones of parasite used [19]. In addition, hemolytic bacteria isolated from the stomach of *R. prolixus,* and identified as *Serratia marcescens,* killed epimastigotes of *T. cruzi* Y but not the Dm28c strain [14], [16]. The *S. marcescens* lytic activity against *T. cruzi* Y strain is inhibited by D-mannose since the bacteria possess mannose-sensitive fimbriae that mediate adherence and lysis of trypanosomes [17], [18]. This intimate interaction of gut microorganisms with parasites emphasizes both the complexity of the triatomine gut environment and the importance of the maintenance of gut homeostasis since many bacteria, fungi and parasites, such as *T. cruzi*, survive and develop in the digestive tract without killing the vector host [7], [8], [15], [19].

One remarkable result of infecting *R. prolixus* with *T. cruzi* Dm28c was the significant decrease in the vector gut microbiota. This was probably related to increases in the antibacterial and PO activities in the gut of infected insects. It is likely that *T. cruzi* Dm28c succeeds in completing development and successfully establishes infection in the vector as a consequence of these changes. In mosquitoes too there is a correlation between microbiota and parasite development [24], [25]. Challenging *Anopheles stephensi, An. gambiae,* and *An. albimanus* with bacteria significantly reduces oocyst infection rates and densities in *Plasmodium falciparum*-infected mosquito cohorts [24]. Subsequently, studies demonstrated that *An. gambiae* microbial flora up-regulates immune genes related to anti-*Plasmodium* factors and in microbe-free aseptic mosquitoes there is an increase in susceptibility to *Plasmodium* infection [25]. Also, in Tsetse flies, bacterial infection enhanced immunity in insects and blocked the trypanosome's ability to establish infections, indicating that bacteria can act to block parasite transmission by up-regulation of some immune responsive genes [26].

Pre-treating the insects with antibiotics before infecting with *T. cruzi* clone Dm28c demonstrates the influence of the insect's microbiota on parasite infection, since a reduced microbiota population caused an increase in parasite numbers. Similarly, in *An. gambiae,* antibiotic-treated insects became more susceptible to *P. falciparum* infection compared with the control septic mosquitoes [25]. In addition, the immune reactions of *R. prolixus* treated with antibiotics were altered in consequence of the higher parasitemia or the reduced microbiota. Thus, antibacterial activity was higher in insects treated with antibiotics and infected with parasites in comparison to both control and infected control insects. However, both nitrite/nitrate production and prophenoloxidase were decreased in insects receiving antibiotic alone and in those given antibiotic and then infected with *T. cruzi* Dm28c.

Regarding nitric oxide (NO) production, a small but significant decrease was observed in the levels of nitrite/nitrate in insects infected with *T. cruzi* Dm28c. It is generally recognized that NO reactions together with oxygen intermediates and derivatives have antiparasitic effects [27]–[29]. Inducible NO has been shown to modulate *R. prolixus* responses to infection by *T. rangeli* in the hemolymph [30] and also demonstrate that NO regulates too the infection by *T. cruzi* in *Rhodnius* [31]. Whitten *et al.* [31] hypothesized that NO synthase gene expression and NO production may be modulated differently by *T. rangeli* and *T. cruzi* infections in various tissues of *R. prolixus*. Their findings showed that in *R. prolixus* infected with *T. cruzi* presented high nitrite concentration in the crop after 1-2 days of blood feeding but nitrite levels decreased after 2 weeks of parasite infection [31]. Our results of low nitrite and nitrate production in the crop of *R. prolixus* infected by *T. cruzi* after nine days of infection are in agreement with these data and demonstrate the complexity of the modulation of NO in this insect. Alterations in NO-derived substances or NOS expression in response to infection have also been described in mosquitoes infected with *Plasmodium* sp [32], [33].

Particularly interesting too was the significant enhanced antibacterial activity and high PO levels in the anterior midgut at day nine after *T. cruzi* Dm28c infection as these paralleled decreases in the microbiota population at this time. The presence of PO in the gut epithelial membrane of *Rhodnius prolixus* with higher levels in the anterior midgut was also identified by Genta *et al.* [22]. A previous report has also shown that two strains of *T. rangeli* parasites can differentially activate the prophenoloxidase system in hemolymph *in vivo* and that this activation may be involved in modulation of the infections [31]. In the present study, a non-invasive parasite, *T. cruzi* Dm28c, stimulates the PO activity in the digestive tract but the parasite population is refractory to killing and completes development in the insect vector. Therefore, the PO, at the concentration recorded, may not significantly inhibit parasite growth, but may differentially kill bacteria that may potentially lyse and compete with the parasites.

Regarding the role of the enhanced antimicrobial activity recorded for the *T. cruzi* Dm28c-infected *R. prolixus,* triatomine vectors have been shown to produce endogenous antimicrobial peptides (defensin and lysozyme) in the gut/fat body following infection with *T. cruzi* or bacteria [34], [35]. Waniek *et al*. [36] demonstrated a marked increase of a defensin transcript in *Triatoma brasiliensis* in response to *T. cruzi* infection and suggested a potential function of gut defensin in parasite population control. Insect antibacterial peptides are often described as molecules that kill bacteria and fungi infections and, as such, they potentially could protect *T. cruzi* from these microorganisms. Direct killing activities have been recorded for some antibacterial compounds against parasites [12], [35]. In these studies, however, high concentrations of heterologous antibacterial peptides have often been used in *in vitro* experiments and *T. cruzi* in the insect vector might never be exposed to such high levels of these compounds or these activities may be affected by gut digestive protease.

Even more significant is the recent description of a novel antimicrobial peptide, prolixicin, from *R. prolixus* with activity against both Gram-negative and Gram-positive bacteria but with no toxicity for *T. cruzi* [37]. This factor may well explain, in the present work, the elevated levels of antibacterial activity recorded in *T. cruzi* infected *R. prolixus* with the differential capability of killing the midgut microbiota but not the *T. cruzi* parasites.

In experimental infections, not all *T. cruzi* strains succeed to grow and complete development in all species of triatomines [15]. Therefore, to test the hypothesis that the parasites infection success is closely related to the microbiota population, we infected *R. prolixus* with *T. cruzi* Y strain which is incapable of completing development in *Rhodnius* digestive tract [14], [16]. The results showed, in contrast to *T. cruzi* Dm28c that *T. cruzi* Y strain could not infect *R. prolixus* and also failed to reduce the microbiota population of the gut. Even when the microbiota population was reduced by treating the insects with antibiotics, *T. cruzi* Y strain did not successfully infect the insects gut. It therefore seems that the parasite infection depends on both the parasite's ability to diminish the bacteria population, which may have trypanolytic activity, and also to modulate the insect's immune reactions.

The present study demonstrates that the activation of PO and antibacterial activities, and the reduction of NO, are related to decreases in the microbiota and increases in *T. cruzi* Dm28c density in the gut. These data, together with previous studies showing other factors that can influence the establishment of parasite infection, demonstrate the molecular complexity involving the interactions of *T. cruzi* with *R. prolixus*.

## Materials and Methods

Parasite infection, microbiota population and immune responses in *R. prolixus* 5^th^-instar nymphs digestive tract were analyzed from 5 to 29 days after treatment.

### Ethics Statement

*Defibrinated rabbit blood was provided by the Laboratory Animals Creation Center (Cecal) which breed and maintain animals for the laboratory. All research programs in Fiocruz respect the guidelines of the Ethics Committee on Animal Use (Ceua) composed by Fiocruz researchers and external consultants.*

### Insects treatment

Fifth-instar *Rhodnius prolixus* nymphs were obtained from a colony reared and maintained in our laboratory at a relative humidity of 50–60% and at 27±2°C. After molting, insects starved for 15–20 days and weighing 35.2±3.4 mg, were randomly chosen and then fed with defibrinated rabbit blood through a membrane feeding apparatus [38]. A control group (C) was fed on blood alone and the infected groups on blood containing 1×10^7^ *Trypanosoma cruzi* Dm28c clone/ml of blood (CC) or Y strain epimastigotes (Y). Insect vector infection by artificial blood feeding is successful when using the epimastigote parasite form which is naturally encountered inside the midgut and capable of adhesion to the gut epithelial cells [9], [16]. In some experiments, groups of insects were fed on blood containing a mixture of three different antibiotics (A), gentamicin, penicillin and streptomycin, with final concentrations of 450, 300 and 300 µg/ml of blood, respectively. Another group of insects were fed on blood containing these antibiotics together with 1×10^7^ *T. cruzi* Dm28c epimastigotes/ml of blood (AC). Only fully engorged insects were used in experiments, which fed upon 180.5±22.1 mg of blood that represents approximately 2.0±10^6^ *T. cruzi* Dm28c epimastigotes/infected insects. All insects were raised and maintained as previously described [39].

### Parasites

*Trypanosoma cruzi* Dm28c clone and Y strain parasites are maintained in our laboratory and grown in a brain heart infusion (BHI, DIFCO) culture medium supplemented with 10% heat-inactivated fetal calf serum at 28°C, according to Azambuja and Garcia [38]. The epimastigotes (99% purity) were obtained from the log-growth phase of the parasites (present up to day 7 of cultivation) [40]. To determine parasite infection in insects, the whole digestive tract was homogenized in 1 ml of sterile phosphate saline buffer (PBS) and parasites counted directly in a hemocytometer.

### Microbiota – Colony Forming Unit (CFU) assay

The microbiota of *R. prolixus* digestive tract were assessed by counting colony forming units (CFU) that grew in brain heart infusion agar (BHI agar). The entire digestive tract was dissected in sterile conditions after different days of treatments and homogenized in 1 ml of sterile phosphate saline buffer (PBS). Samples were then immediately transferred to ice, diluted 10^−5^, 10^−7^ or 10^−9^, with PBS, 20 μl aliquots spread onto BHI agar plates and then incubated overnight at 30°C and CFUs subsequently counted. A PBS aliquot was also plated as a control to guarantee sterility. The insects, *R. prolixus*, maintained in our colony present a natural microbiota flora which contains different bacteria species including *Serratia marcescens* strain. These insects were used in our experiments without infecting with other bacteria.

### Antibacterial assays

To analyze the antibacterial activity in anterior and posterior midguts, tissues were dissected and disrupted in 0.1% Triton X-100 (v/v) at 4°C. The samples were centrifuged at 8,000 g for 1 min at 4°C. Aliquots were taken from the supernatant for each assay, as described below.

#### Zone of inhibition (ZI) assay

Aliquots of 20 µl from the *R. prolixus* gut samples collected and prepared, as described above, were kept frozen at −20°C for no more than 2 weeks. The samples (5 µl) were dispensed in triplicate on to sterile filter paper discs, arranged on the surface of soft agar plates and incubated at 37°C for 18 h. Plates were prepared using 1% agarose in liquid culture medium (BHI) containing streptomycin (100 μg/ml) and ampicillin (80 μg/ml) and *Escherichia coli* D31 incorporated into the agar. Antibacterial activity of the gut samples was recorded as diameters (cm) of the growth inhibition zones of *E. coli* D31 on the agarose plates. Preliminary tests, undertaken to assess the antibacterial activities of anterior and posterior midgut samples at different days after treatment, showed the highest activity (ZI) in the anterior midgut nine days after feeding. Therefore, all experiments investigating the insects immune responses were standardized using samples of the anterior midguts (tissues and contents) taken nine days after treatments.

#### Turbidometric (TB) assay

The TB assay was modified from Thomas *et al*. [41] and Bexfield *et al.* [42]. Anterior midgut samples, nine days after treatment, as described above, were centrifuged at 8,000 g for 10 min at 4°C and 70 µl of supernatant transferred into tubes containing 630 µl of Milli-Q water. All samples were filter sterilized and frozen at −20°C. The assay involved incubating the midgut samples with 1% peptone plus the test bacteria, *Escherichia coli* k12. *E. coli* k12 were grown in 20 ml tryptone soy buffer (TSB) for 17 h at 30°C with oscillation and then 100 µl of the bacteria were transferred to 10 ml of TSB and incubated for a further 4 h. The bacteria were then washed in PBS and diluted in TSB to a final concentration of 1×10^5^ cells/ml. Fifty microlitres of midgut sample were then incubated with 10 μl of bacterial suspension in triplicate in the wells of a sterile flat-bottom, 96-well microtiter plate (Nunc, Fisher Scientific UK, Leicestershire, UK). Control wells without midgut extracts contained (1) bacteria, peptone and antibiotic [streptomycin (100 μg/ml) and ampicillin (80 µg/ml)]; (2) bacteria and peptone; or (3) peptone alone. The optical densities were measured at 550 nm (OD_550_) during 12 h incubation at 37°C and read at hourly intervals from time zero. All data points were subsequently blanked against time zero to account for the opacity of the midgut samples and then the bacteria *E. coli* k12 readings were subtracted from all sample readings and multiplied by 100.

Anterior midgut samples (50 µl), from control insects (C) and infected insects (CC), were also incubated with 10 µl of *E. coli* k12 (1×10^5^ cells/ml) and 10 µl of peptone, in tubes at 37°C, concurrently with the TB assay. At different time during incubation, samples were plated onto BHI-agar to compare the bacterial growth with the readings in the TB assay.

### Phenoloxidase (PO) activating assay

Anterior midgut samples freshly prepared were collected at nine days after insect treatments, as described above, and 10 µl of supernatant were transferred into tubes containing 90 µl of Milli-Q water. To determine the phenoloxidase (PO) activity in anterior midgut portions, all homogenates used for enzymatic assays were freshly prepared and PO activity was determined by measuring the production of dopachrome from DOPA [23], [43], [44]. The assay was prepared by incubating 25 μl of sample in triplicate with 10 μl of 10 mM sodium cacodylate pH 7.4 containing 10 mM CaCl_2_ buffer and 25 μl of a saturated solution of DOPA (4 mg/ml). The absorbance at 490 nm was measured in a microplate reader for 120 min at 37°C with readings taken every 15 min. The values of enzymatic activity presented are expressed as abs/mg protein and refer to specific PO activity by protein concentration in anterior midgut homogenates multiplied by 100.

### Nitrite and nitrate determinations

The anterior midgut samples were freshly prepared and collected at nine days after insect treatments, as described above, and 10 µl of supernatant were diluted in 90 µl of Milli-Q water. Nitrate and nitrite contents of samples, which can be indicative of reactive nitrogen intermediate (RNI) metabolism, were analyzed following the manufacturer's instructions using the Griess Reagent System Assay Kit (Promega, WI, USA) [45], and absorbance of the product was measured at 550 nm. Nitrite and nitrate contents were quantified as µmoles using a range of sodium nitrate standards and the specific activity was calculated mg/ml of protein concentration in anterior midgut samples.

### Protein determination

Protein content of samples was quantified with a protein assay kit (BCA* Protein Assay Reagent, Pierce, USA) using bovine serum albumin (BSA) standards.

### Statistical analyses

The results were analyzed with GraphPad Prism 5 using 1 Way ANOVA or unpaired T test, or Mann Whitney test (nonparametric test) depending on the data distribution and number of treatments. Data are reported as mean ± standard error (SE) or as individual values with medians for parasite and microbiota populations. Differences among groups were considered not statistically significant when p>0.05. Probability levels are specified in the text and Figure legends.
